# The Scarlet Alchemy of Survival: Integrated Transcriptomic and Metabolomic Analysis of Leaf Coloration in Endangered *Parrotia subaequalis*

**DOI:** 10.3390/plants14152345

**Published:** 2025-07-29

**Authors:** Lifang Zhang, Aya Hafsi, Xianting Wang, Chenyun Zhang, Zedong Lang, Mingjian Yu, Yanming Fang

**Affiliations:** 1 Zhejiang Academy of Forestry, Hangzhou 310023, China; zzllffmeng@163.com; 2 College of Life Sciences, Zhejiang University, Hangzhou 310058, China; ayahafsee@gmail.com (A.H.); fishmj@zju.edu.cn (M.Y.); 3 Anji Hynobius amjiensis National Nature Reserve, Huzhou 313399, China; wangxianting2024@126.com (X.W.); 15257222607@163.com (Z.L.); 4 College of Resources and Environmental Sciences, Nanjing Agriculture University, Nanjing 210095, China; zcy275422@163.com; 5 Key Laboratory of Subtropical Forest Biodiversity Conservation, State Forestry Administration, Co-Innovation Center for Sustainable Forestry in Southern China, College of Biology and the Environment, Nanjing Forestry University, Nanjing 210037, China

**Keywords:** leaf color variation, pigmentation mechanisms, different growth stages, leaf phenotypic, metabolome, transcriptome, *Parrotia subaequalis*

## Abstract

*Parrotia subaequalis* is a rare and endangered deciduous tree native to China, valued for its vibrant autumn foliage and ornamental appeal. Its leaves exhibit striking coloration, ranging from red to yellow and purple, yet the physiological and molecular mechanisms behind this variation remain poorly understood. Here, we combined transcriptomic, metabolomic, and physiological analyses to investigate pigment changes within the yellow leaf phenotype of *P. subaequalis*. Our findings revealed significant differences in gene expression and metabolic profiles between yellow and green leaves, particularly in starch and sucrose metabolism, photosynthesis, and carbon metabolism. Yellow leaves exhibited reduced photosynthetic capacity and carotenoid levels, alongside increased D-glucose concentration. These findings suggest that visible color transitions are likely driven by coordinated changes in carbohydrate metabolism, photosynthetic function, and organic compound accumulation. This study provides novel insights into the molecular and physiological mechanisms governing leaf pigmentation in an endangered tree, with useful information relevant to their conservation and sustainable utilization.

## 1. Introduction

Leaf color variation is a readily observable and visually striking phenotypic trait in many temperate species, especially during seasonal transitions [1,2,3]. These color changes result from altered concentrations of pigments such as chlorophylls, carotenoids, and anthocyanins, and they often mirror the plant’s response to environmental stresses and selective pressures [4,5,6]. Moreover, color change is often accompanied by changes in leaf tissue, cellular organization, and pigment distribution [7,8].

While ecological and physiological studies have linked leaf pigmentation to environmental conditions, the underlying molecular mechanisms governing these changes remain poorly understood, especially in non-model and rare or ecologically restricted species. This knowledge gap limits our understanding of how pigmentation traits contribute to adaptation or stress tolerance beyond a few well-studied taxa. Advances in transcriptomics and metabolomics now make it possible to investigate these questions more deeply. Transcriptome sequencing enables comprehensive profiling of gene expression patterns, identifying genes involved in pigment synthesis pathways and their regulatory networks [9,10,11,12,13]. For example, differentially expressed genes (DEGs) related to photosynthesis, secondary metabolites, and environmental adaptation to stress have been identified as important regulators of leaf pigmentation change [14,15,16,17]. Likewise, metabolite profiling can reflect the pigment abundance, and the physiological state of a plant can confirm the accumulation of pigments such as anthocyanins and carotenoids that drive visible coloration [18]. Disruptions in leaf pigment biosynthesis are often associated with broader morphological and physiological changes, including altered hormonal balance, impaired carbon assimilation, and reduced stress tolerance [19,20,21,22,23].

Many of these pigment differences are driven by transcriptional regulation of biosynthetic genes. For instance, the anthocyanin biosynthesis is tightly regulated at the transcriptional level by the evolutionarily conserved MYB-bHLH-WD repeat (MBW) complex [24,25,26]. Manipulating the expression of these transcription factors (TFs) can significantly alter anthocyanin production [27,28] and, by extension, leaf color and stress tolerance. Integrating transcriptomic and metabolomic analyses offers a comprehensive view of these regulatory networks, enabling the identification of key genes, regulatory pathways, and metabolic changes involved in pigment biosynthesis, and clarifying their links to plant developmental and ecological processes [29,30,31].

**Figure 1:**
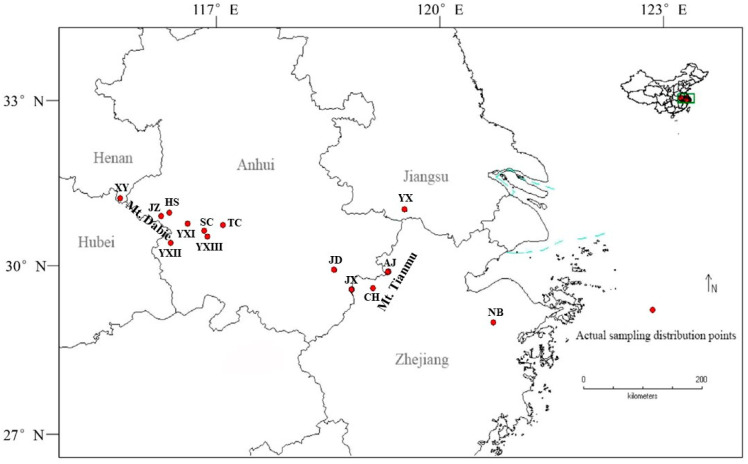
Distribution map of *Parrotia subaequalis* populations across eastern China in 2025. The red circles represent the extant population sites.

Building on this understanding of pigment regulation, we focused on *Parrotia subaequalis*, which is a rare and endangered species endemic to China. Widely appreciated for its vivid seasonal foliage as an ornamental tree used in landscaping, this species displays striking leaf color variation [32,33,34] that may reflect adaptation to heterogeneous microhabitats across its fragmented range of only 14 populations known form Anhui, Jiangsu, Henan, and Zhejiang provinces [35,36] (Figure 1, Table 1 and Table 2). Despite its ecological value and conservation status, the molecular mechanisms underlying its pigment diversity remain poorly understood.

In this study, we focused on 14 populations of *P. subaequalis* [37], which were grown in the common garden of Anji Lingfeng Temple Forest Farm (119°38′22.1″ E, 30°35′55.4″ N) in Zhejiang Province. After the leaf color observation (Table 1), we focused on the YX population, which consistently exhibited distinct and well-defined seasonal green to yellow color transitions. We, therefore, selected this population for detailed phenotypic comparison with other populations, and physiological, transcriptomic, and metabolomic analyses across the four discoloration stages (Figure S1).

We hypothesized the following: (i) key pigments (e.g., total chlorophyll, chlorophylls a and b, carotenoids, and lutein) and leaf traits vary across color stages and different populations, respectively; (ii) differentially expressed genes (DEGs) and differentially accumulated metabolites (DAMs) involved in pigment biosynthesis would be enriched in colored leaves; (iii) photosynthetic parameters and metabolic indicators would be reduced in colored leaves relative to green leaves. Our findings aim to provide critical insights into the molecular mechanisms governing leaf pigment variation in this critically endangered species, contributing to its field conservation strategies and sustainable utilization. By linking molecular and physiological patterns to visible phenotypes, this work contributes to understanding the adaptive biology of *P. subaequalis* and supports its conservation and horticultural potential.

## 2. Results

### 2.1. Phenotypic Variation in Leaves Across Different Populations

**Figure 2:**
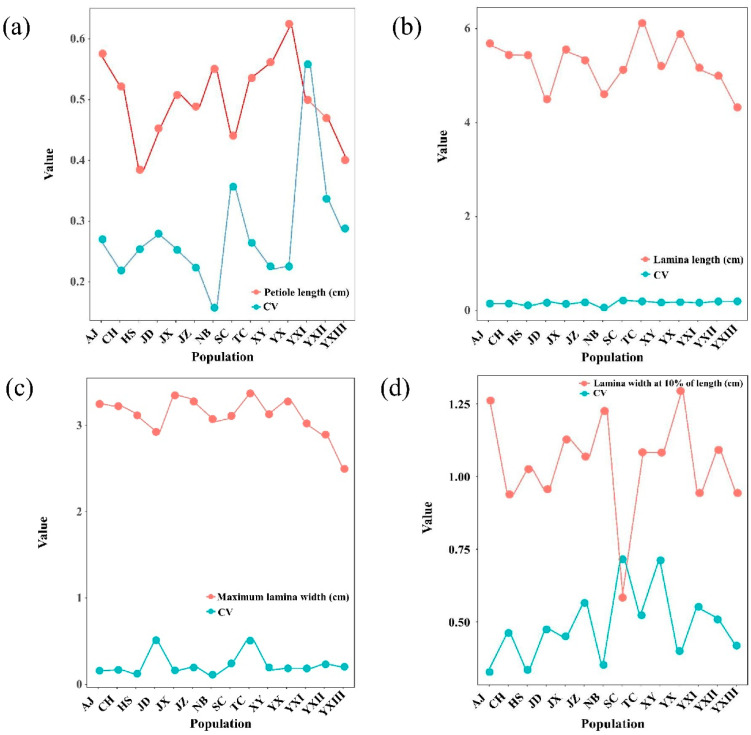
Mean values and CVs of 4 leaf phenotypic traits across 14 populations of *Parrotia subaequalis*. (**a**) Petiole length; (**b**) lamina length; (**c**) maximum lamina width; (**d**) lamina width at 10% of length.

**Figure 3:**
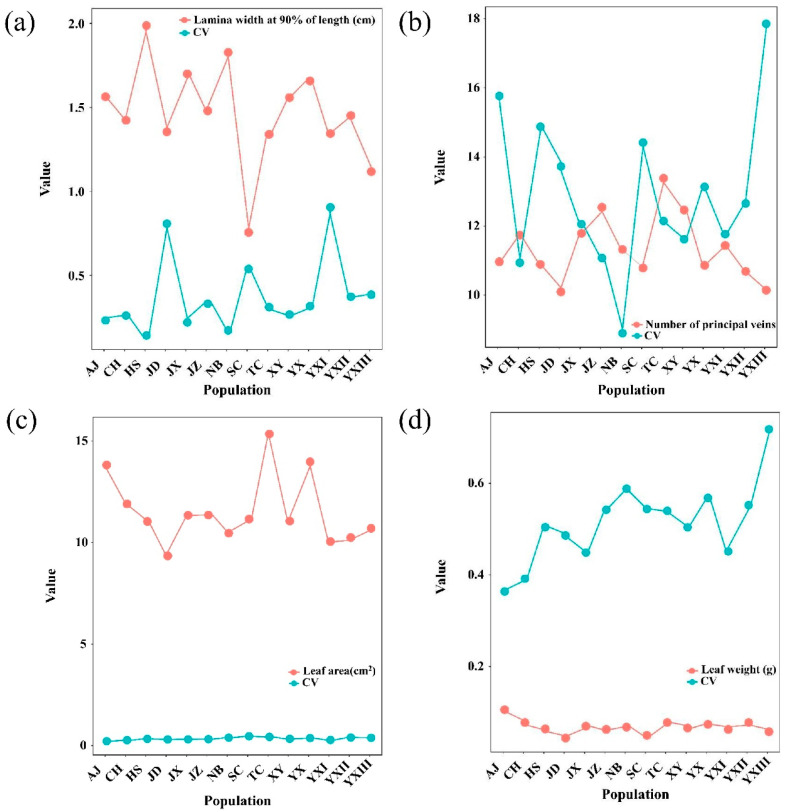
Mean values and CVs of 4 leaf phenotypic traits across 14 populations of *Parrotia subaequalis*. (**a**) Lamina width at 90% of length; (**b**) number of principal veins; (**c**) leaf area; (**d**) leaf weight.

After four years of growth, significant leaf phenotypic variations were observed among 14 provenances from 14 populations of *P. subaequalis* (Table 3 and Table 4). The coefficients of variation for petiole length and lamina were all higher than the overall average, while leaf length and number of primary leaf veins were all lower than the overall average. Several populations (JD, SC, YXI, and XY) had the highest average CVs, while others (NB, HS, JX, and YX) had lower average CVs (Figure 2 and Figure 3). Generalized linear regression revealed varied effects among populations based on phenotypic traits. Among the six phenotypic traits, maximum lamina width in the JD population was significantly different from that in other populations (*p* < 0.001). Lamina width at 10% of length also varied significantly in the JZ, SC, and XY (*p* < 0.001), as well as in the TC, YXI, and YXII (*p* < 0.05) compared to those in other populations. Furthermore, lamina width at 90% of length in the JD, SC, and YXI populations (*p* < 0.001) differed from that in other populations (Tables S1–S6).

Petiole length and lamina width (measured at 0.1 of its length) were greatest in the XY population, while the TC population exhibited the longest lamina and the widest lamina overall. In contrast, the YXIII population consistently displayed the smallest leaf dimensions. The number of principal veins differed significantly among populations, with the highest average observed in JZ and the lowest in JD (Table 5; Figure 2 and Figure 3). Leaf area and leaf weight also varied, with the YX population showing notably large leaf area values, whereas the JD population had the lowest (Figure 3c). Leaf weight was highest in AJ and lowest in JD populations (Figure 3d). These results suggest substantial population-level differentiation in leaf morphology, likely reflecting both genetic and environmental influences.

### 2.2. Differentiation of Gene Expression of Leaf Pigment Change

**Figure 4:**
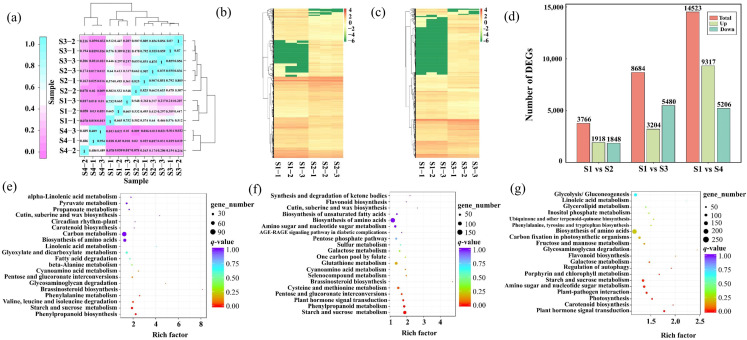
Differentially expressed genes (DEGs) and KEGG pathway enrichment across four leaf developmental stages. (**a**) Pearson correlation matrix among 12 samples (4 stages × 3 replicates). (**b**,**c**) Hierarchical clustering of gene expression across samples; red = high expression, green = low expression. (**d**) Number of total, up-regulated, and down-regulated DEGs. (**e**–**g**) KEGG pathway enrichment of DEGs in comparisons of S1 vs. S2, S1 vs. S3, and S1 vs. S4. The *x*-axis indicates the rich factor; dot color and size correspond to q-value and gene count, respectively.

To further compare the gene expression profiles across the four leaf development stages of *P. subaequalis*, pairwise comparisons of gene expression levels and differentially expressed genes (DEGs) were identified. High-throughput sequencing of leaf transcriptomes across four developmental stages yielded between ~20 and ~33 million clean reads per sample, with Q20 and Q30 values exceeding 96% and 91%, respectively, indicating high data quality (Table S7). Moreover, replicate samples were highly correlated, supporting the uniformity of RNA-seq data (Figure 4a). Gene expression during each stage of growth was highly variable (Figure 4b,c). Expression levels and annotation for all genes are shown in Table S8.

Differentially expressed genes were identified through pairwise comparisons of the earliest developmental stage (S1) with subsequent stages (S2, S3, and S4) (Figure 4d) (Table S9). The number of DEGs increased progressively from S1 vs. S2 to S1 vs. S4, with the largest transcriptomic shift observed between stages S1 and S4. This substantial change may reflect regulatory transitions involved in pigment accumulation and the development of photosynthetic capability. Gene Ontology (GO) analysis assigned a total of 29,469 unigenes to biological processes, cell components, and molecular functional classes. The clusters of orthologous groups (COGs) of the proteins database annotation allocated 20,171 unigenes into 26 COG categories. The most abundant functional categories included general function prediction, translation-related processes, and carbohydrate metabolism, reflecting core metabolic and regulatory activity during leaf development.

KEGG enrichment analyses revealed dynamic shifts in gene expression across stages. In the S1 vs. S2 comparison, DEGs were primarily associated with phenylpropanoid biosynthesis, starch and sucrose metabolism, leucine and isoleucine degradation, and phenylalanine metabolism. Up-regulated genes were mainly associated with ribosome activity, carbon metabolism, and glyoxylate and dicarboxylate metabolism, while down-regulated genes were linked to phenylpropanoid biosynthesis and starch and sucrose metabolism (Figure 4e). In the S1 vs. S3 comparison, DEGs were enriched in starch and sucrose metabolism, phenylpropanoid biosynthesis, and plant hormone signal transduction. Up-regulated genes were associated with ribosome and glutathione metabolism. Down-regulated genes were linked to plant hormone signal transduction, starch and sucrose metabolism, phenylpropanoid biosynthesis, and photosynthesis (Figure 4f). In the S1 vs. S4 comparison, DEGs were primarily involved in starch and sucrose metabolism, amino sugar and nucleotide sugar metabolism, and photosynthesis. Among these, up-regulated DEGs were associated with carbon metabolism, whereas down-regulated DEGs were associated with starch and sucrose metabolism, plant–pathogen interaction, and photosynthesis (Figure 4g). These results suggest that leaf pigment change may result from reduced starch and sucrose metabolism and photosynthesis.

**Figure 5:**
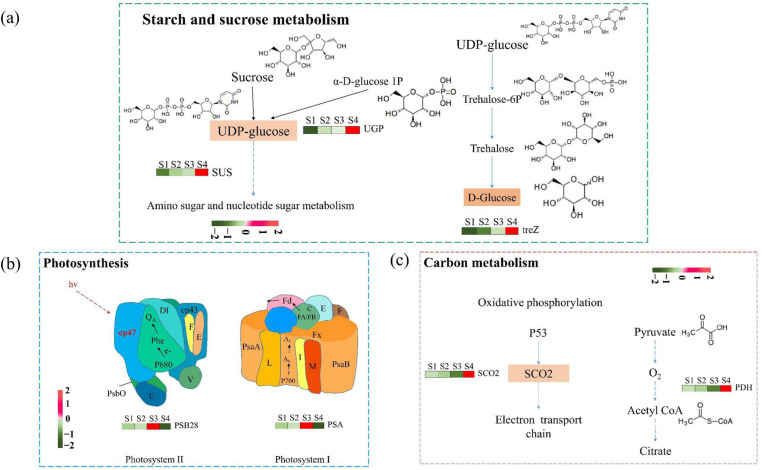
Differentially expressed genes (DEGs) mapped to key metabolic pathways associated with leaf color change in *Parrotia subaequalis*. Panels show DEGs involved in (**a**) starch and sucrose metabolism, (**b**) photosynthesis, and (**c**) carbon metabolism during four developmental stages (S1–S4). Gene expression levels are visualized using log_2_ fold-change heatmaps across the stages. In starch and sucrose metabolism (**a**), up-regulated genes included *SUS* (sucrose synthase), *UGP* (UDP-glucose pyrophosphorylase), and *treZ* (malto-oligosyltrehalose trehalohydrolase), corresponding to increased D-glucose accumulation. In photosynthesis (**b**), expression changes were observed in *PSB28* (photosystem II reaction center protein 28) and *PSA* (photosystem I subunit A), indicating shifts in light-harvesting complex activity. In carbon metabolism (**c**), *SCO2* (cytochrome c oxidase synthesis homolog) and *PDH* (pyruvate dehydrogenase) were differentially expressed, reflecting modifications in mitochondrial energy pathways and carbon flux. Red and green colors represent up-regulation and down-regulation, respectively, across leaf stages S1 to S4.

We further analyzed DEGs involved in starch and sucrose metabolism, photosynthesis, and carbon metabolism. Notably, *SUS*, *UGP,* and *treZ* were down-regulated in the S4 stage compared with the S1, S2, and S4 stages, potentially reducing starch and sucrose biosynthesis (Figure 5a). In photosynthesis, *PSA* and *PSB28* were down-regulated in the S4 stage compared to the S1, S2, and S3 stages, which may affect the activity of photosystem I and photosystem II (Figure 5b). In carbon metabolism, *PDH* and *SCO2* were up-regulated in the S4 stage compared with the S1, S2, and S3 stages (Figure 5c). Additionally, we predicted that several transcription factors (TFs), including MYB-related, bHLH, WD40, WRKY, bZIP, and NAC may regulate the pigment changes in yellow leaves (Figure S2). These TFs are mostly associated with responses to changes in leaf pigmentation.

### 2.3. Metabolome Profiling of P. subaequalis

**Figure 6:**
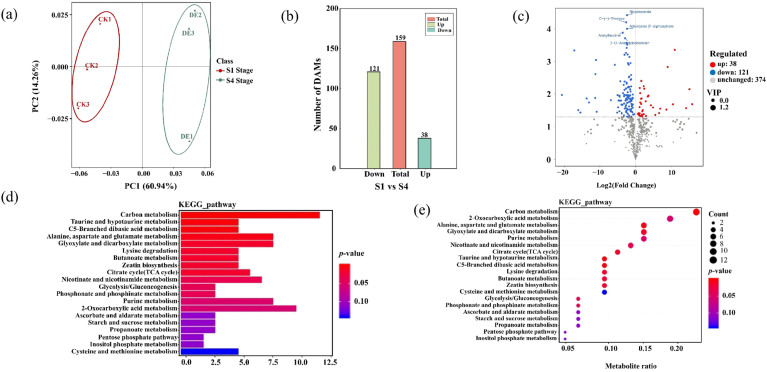
Metabolomic profiling of *Parrotia subaequalis* leaves at two developmental stages (S1 vs. S4). (**a**) Pearson correlation matrix among six samples (two stages × three biological replicates), showing reproducibility within groups. (**b**) Total number of differentially accumulated metabolites (DAMs), including up-regulated and down-regulated compounds. (**c**) Volcano plot showing distribution of DAMs based on fold change and statistical significance. (**d**,**e**) KEGG enrichment analysis of DAMs between stages S1 and S4. The *x*-axis indicates metabolite ratios; dot color represents –log_10_ (*p*-value), and dot size corresponds to the number of enriched metabolites.

Since the DEGs identified in the transcriptome were highly enriched in starch and sucrose and photosynthesis metabolism-related pathways, a subsequent metabolomics analysis was performed to uncover the metabolic distinctions among the four stages related to leaf pigmentation. Principal component analysis (PCA) results revealed significant segregation between the two stages, particularly highlighting apparent metabolic differences, especially in the S4 stage compared with the A stage (Figure 6a).

DAMs were screened based on their accumulation and annotation (Table S10). A total of 159 differentially accumulated metabolites (DAMs) were identified between stages S1 and S4, with the majority being down-regulated (Figure 6b,c; Table S11). This indicates a general decline in metabolite abundance during later stages of leaf development. In S1 vs. S4 stages, the main function of DAMs were linked to carbon metabolism, alanine, aspartate and glutamate metabolism, glyoxylate and dicarboxylate metabolism, and citrate cycle (Figure 6d). Finally, the KEGG enrichment analysis of S1 vs. S4 stages revealed DAMs to be mainly associated with the carbon metabolism, alanine, aspartate and glutamate metabolism, glyoxylate and dicarboxylate metabolism, and taurine and hypotaurine metabolism (Figure 6e; Tables S12 and S13).

Given the significantly different expression of the carbon metabolism pathway in the S1 stage compared to the S4 stage (Figure 6d,e), a detailed analysis of metabolites within this pathway was conducted. Nicotinamide, D-(-)-threose, adenosine 5′-diphosphate, alanylleucine, and 3-o-acetylpinobanksin were significantly down-regulated in S1 vs. S4 stages. Conversely, D-glucose levels were up-regulated in the S1 vs. S4 stages. Carbon metabolism is known to play a critical role in the synthesis, degradation, and interconversion of photosynthetic assimilates in plants, and DAMs in carbon metabolism enhance D-glucose contents, potentially leading to leaf color changes.

### 2.4. Correlated Transcriptome and Metabolome Analyses of Leaf Pigment Change

**Figure 7:**
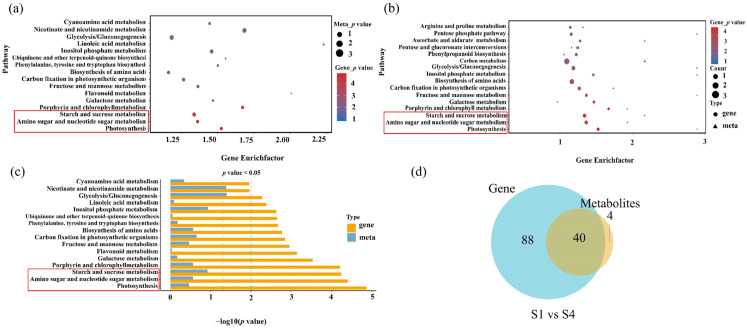
Correlation analyses of transcriptome and metabolome. (**a**) Significantly enriched KEGG pathways of DAMs in metabolome (the red box). (**b**,**c**) Significantly enriched KEGG pathways of DAMs and DEGs in S1 vs. S4 stages (the red box); (**d**) a Venn diagram showing differentially expressed gene pathways and differentially abundant metabolite pathways. The *x*-axis indicates the gene ratio or metabolite ratio. The *y*-axis indicates the KEEG pathway co-enriched in the transcriptome and metabolome. The color and size indicated the –log_10_ (*p*-value) and metabolite number as shown on the right.

A correlation analysis of the transcriptome and metabolome was performed to investigate the relationship between DEGs and DAMs associated with the leaf pigment changes. Pathways including starch and sucrose metabolism, amino sugar and nucleotide sugar metabolism, and photosynthesis were jointly enriched in both datasets in the S1 and S4 stages comparisons (Figure 7). Key regulatory genes and corresponding metabolites within these pathways were examined to identify coordinated transcriptional and metabolic responses.

**Figure 8:**
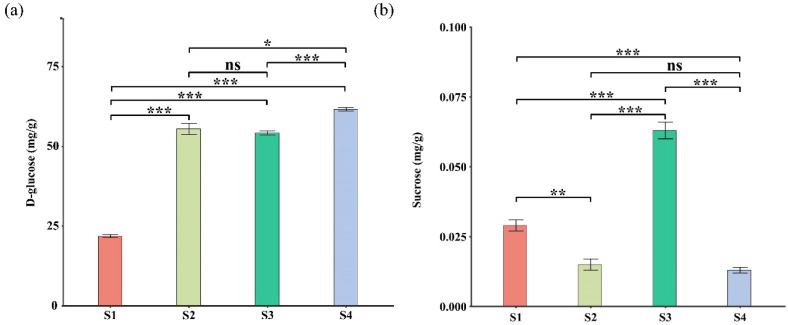
Organic content of each leaf growth stage of *P. subaequalis*. (**a**) D-glucose; (**b**) sucrose. Data represent the means ± SEM from least biological replicates, * *p* < 0.05, ** *p* < 0.01, *** *p* < 0.001, by *t*-test, ns, not significant.

Significant changes in starch and sucrose metabolism were observed in gene expression and metabolite content (Table S10). After aligning their sequence IDs to relevant genes, statistical analysis revealed that key regulatory genes involved in starch and sucrose metabolism (e.g., *PGM1*, *PGM2*, *HXK1*, *HXK2*, and *UGPase*) were predominantly up-regulated in S1 vs. S4 stages. This up-regulation resulted in an increase in D-glucose level and a decrease in sucrose content (Figure 8), which ultimately influenced leaf pigmentation. In amino sugar and nucleotide sugar metabolism, most DEGs and DAMs were consistent with those in starch and sucrose metabolism. In the photosynthesis pathway, *NYCL* and *NOL*, which are associated with chlorophyll degradation, were also up-regulated in S1 vs. S4 stage comparison.

To validate the reliability of transcriptome results, four genes were randomly selected for expression analysis. As shown in Figure S3, the gene expression levels were found to be consistent with the patterns observed in the transcriptome data, further corroborating the reliability of our transcriptome findings.

### 2.5. Leaf Pigment Change Enhanced Photosynthetic Pigments Content and Organic Compound

**Figure 9:**
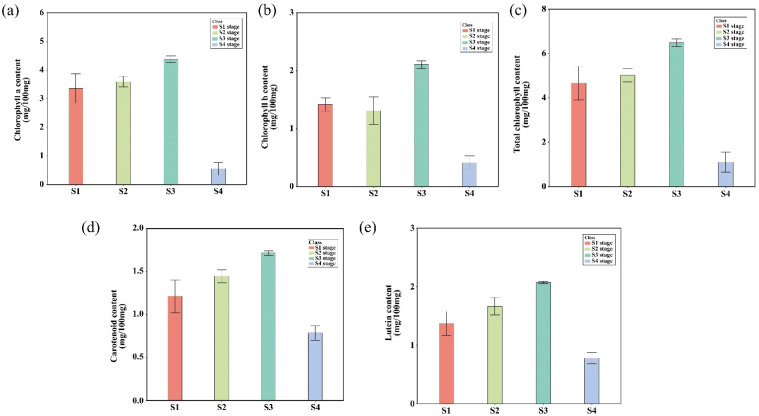
Photosynthetic pigments of each leaf growth stage of *P. subaequalis*. (**a**) Chlorophyll a content; (**b**) chlorophyll b content; (**c**) total chlorophyll content; (**d**) carotenoid content; (**e**) lutein content. Data represent the means ± SEM from at least three biological replicates.

**Figure 10:**
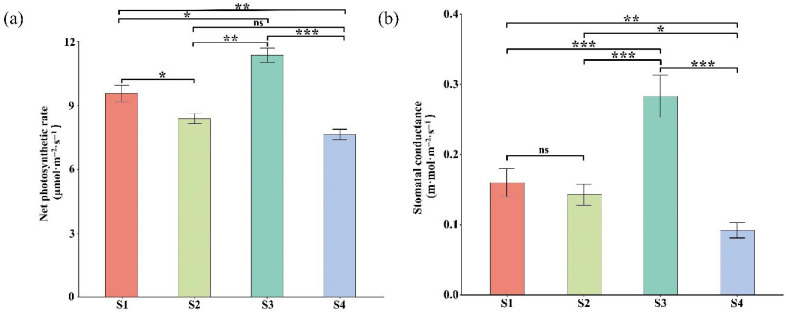
Photosynthetic capacity and pigments from each leaf growth stage of *P. subaequalis*. (**a**) The net photosynthetic rate; (**b**) stomatal conductance. Data represent the means ± SEM from at least three biological replicates; * *p* < 0.05, ** *p* < 0.01, *** *p* < 0.001, by *t*-test, ns, not significant.

DEGs and DAMs related to photosynthesis were found to be interconnected in the S1 vs. S4 stages. To validate the specific changes in these pathways, we compared chlorophyll content, photosynthetic capacity, and glucose content. The content of chlorophyll a (Figure 9a), chlorophyll b (Figure 9b), total chlorophyll (Figure 9c), carotenoid (Figure 9d), and lutein (Figure 9e) were higher in the S1 stage compared to the S4 stage. Additionally, net photosynthetic rate (Figure 10a) and stomatal-conductance significant differences across the four stages (Figure 10b). Stage S3 exhibited the highest photosynthetic capacity, correlating with the production of the highest glucose levels. In contrast, stage S4 had reduced photosynthetic capacity, which was associated with lower glucose levels, consistent with the observed down-regulation of photosynthesis-related genes and metabolites (Table S14).

## 3. Discussion

*Parrotia subaequalis* exhibits notable leaf color diversity and morphological variation across its natural range. Our four-year common garden study revealed significant differences in eight leaf traits among 14 geographically distinct populations, suggesting strong phenotypic plasticity and potential for local adaptation. Some populations showed high within-population variability, while others were more morphologically uniform. The YX population—although lower in overall trait variability—displayed consistent seasonal transitions from green to yellow leaves, making it an ideal model for investigating the molecular basis of pigment change. Leaf pigment change is a complex physiological and biochemical process that plays an important role in plant adaptation to the environmental conditions and the regulation of photosynthesis. This study aimed to test three hypotheses regarding the molecular and physiological mechanisms underlying leaf color variation. The results supported our first hypothesis: colored leaves exhibited reduced net photosynthetic rate and stomatal conductance, consistent with diminished photosynthetic efficiency. The second hypothesis was also supported: DEGs and DAMs in colored leaves were enriched in pathways related to starch and sucrose metabolism, photosynthesis, and carbon metabolism. However, the third hypothesis was not fully supported: although D-glucose levels increased in yellow leaves, the content of sucrose was lower than in green leaves.

### 3.1. Phenotypic Variation in Leaves Across P. subaequalis Populations

Both among and within 14 *P. subaequalis* populations, there was a high level of variation in leaf traits. While several traits exhibited quite high levels of variation among populations, others (e.g., leaf length and the number of principal leaf veins) were less variable. Similar patterns have been reported for the phenotypic traits of *Rhododendron taibaiense* and *Paeonia ludlowii* [38,39]. This may be because the stability of upper leaf width is the lowest among leaf phenotypic traits, whereas the number of main veins and the leaf index are relatively stable under variable environmental conditions and have a stable genetic basis.

Within populations, some had very high variance in leaf traits (e.g., JD, SC, YXI, and XY), while others were quite invariable (NB, HS, JX, and YX). One reason for this could be that populations with higher within-population variation have higher habitat heterogeneity, while stable habitats or genetic bottlenecks may constrain phenotypic diversity [29,40,41]. Supporting this observation, the JD, YXI, SC, and XY populations exhibited high environmental variability (e.g., average annual precipitation, altitude, and average annual air temperature), while other populations showed lower environmental values. Notably, within the same region, one population (YX) was lower than the overall average, indicating reduced morphological diversity, while another (YXI) had high variation, possibly reflecting differences between distinct subpopulations within the same region.

The YX population had both relatively long petioles and wide lamina, whereas HS and SC populations were the smallest. However, the leaves from the YX population were not the largest for all traits, such as leaf area and leaf weight. The variability in leaf morphology in *P. subaequalis* is highly variable and likely shaped by a combination of genetic and environmental factors. The unique characteristics of the YX population warrant further investigation to elucidate the specific environmental pressures or genetic adaptations contributing to its distinct leaf morphology. The observed differences between populations underscore the species’ capacity for local adaptation and phenotypic plasticity, which are essential for survival in heterogeneous environments [42,43,44].

Furthermore, the manifestation of yellowing leaves, characterized by yellow leaf, in YX population of *P. subaequalis* likely signifies an ecological adaptation to fluctuating environmental conditions. This occurrence may correlate with responses to environmental stress, as the yellowing can facilitate the optimization resource allocation amidst changing environmental parameters. Additionally, it could act as a photoprotective strategy, mitigating damage caused by excessive sunlight exposure, and assist in modulating the microclimate surrounding the leaf by influencing transpiration rates and temperature [45,46]. Consequently, the yellowing of leaves is intricately connected to environmental adaptation, light protection, and microclimatic influences, thereby bolstering the species’ survival and adaptability within diverse and variable habitats.

### 3.2. The Genes and Metabolites Involved in Leaf Pigmentation of P. subaequalis

The relatively larger leaves in the YX population suggest a more efficient light capture strategy compared to other populations. This adaptation may be crucial for survival and growth in environments with limited light availability. Additionally, the leaves from this population turn yellow in autumn, making them an interesting test case to study leaf pigmentation change. This transition phase offers a clear visual shift and often involves significant alterations in the levels of chlorophyll, carotenoids, and other pigments, providing a valuable opportunity to investigate the underlying biochemical and genetic mechanisms regulating these changes.

By conducting transcriptome and metabolome profiling from distinct developmental stages (S1 to S4 stages), we were able to identify many DEGs involved in starch and sucrose metabolism, amino sugar and nucleotide sugar metabolism, photosynthesis, and carotenoid biosynthesis in S1 vs. S4 stages. Similarly, DAMs were mainly associated with carbon metabolism, aspartate and glutamate metabolism, glyoxylate and dicarboxylate metabolism, and citrate cycle. These findings were consistent with most pathways enriched in the transcriptome analysis. Furthermore, DEGs and DAMs were strongly associated with enriched biological pathways, corroborating previous findings that suggest similar mechanisms regulate leaf pigmentation [47,48,49,50]. Based on our physiological measurements and multi-omics analysis, the leaf pigmentation changes in *P. subaequalis* can be summarized as being driven primarily by two factors: pigment content and type change, and increased accumulation of organic compounds.

Chlorophyll, carotenoid, and lutein contents increased to a peak mid-season and then decreased throughout the growing season. Leaf pigment changes during plant growth and development are critically regulated by pigment type and content [51,52]. Chlorophyll and carotenoids are primary pigments involved in photosynthesis, and their synthesis and degradation significantly impact photosynthesis [53,54,55]. During leaf growth and development, the degradation of chlorophyll and the synthesis of carotenoids regulate pigment changes, causing leaves to display yellow and red hues in autumn [56,57,58,59].

In this study, the contents of chlorophyll, carotenoids, and lutein peaked in the S3 stage compared to the S1, S2 and S4 stages. Contrary to our initial expectations, there was no further increase in pigment content. Chlorophyll and carotenoids are critical photosynthetic pigments, and their synthesis and degradation significantly impact photosynthesis [53,54,55]. The decreased photosynthetic parameters in the S4 stage likely contribute to the observed changes in leaf color. Additionally, the expression of genes such as *PSA* and *PSB28*, which play a decisive role in net photosynthetic rate, was significantly down-regulated in the S4 stage compared to the S1, S2 and S3 stages (Figure S3). Therefore, the results suggest that changes in pigment content and type affect photosynthesis, and weakened photosynthesis, in turn, influences the synthesis of organic compounds.

Several DEGs and DAMs indicate that leaves in the later stage exhibited higher starch and sucrose metabolism. Leaf pigment changes are closely linked to the levels of organic compounds in the leaf, which can impair photosynthetic efficiency. D-glucose, a fundamental sugar produced during photosynthesis, declines as photosynthesis decreases and resources are reallocated, accelerating chlorophyll breakdown and revealing underlying carotenoid pigments, ultimately influencing leaf coloration [60,61,62,63]. In this study, most genes and metabolites involved in starch and sucrose metabolism were up-regulated in the later season, particularly those involved in D-glucose biosynthesis, including *PGM1*, *PGM2*, *HXK1,* and *HXK2*. This suggests a significant accumulation of D-glucose, which may contribute to the observed changes in leaf color from green to yellow in cold weather.

The regulatory mechanisms underlying leaf color variation and phenotypic plasticity in *P. subaequalis* exhibit broad relevance to other temperate deciduous tree species. Many trees, including species within *Acer*, *Fraxinus*, and *Rhododendron*, display comparable seasonal patterns: pigment shifts, reduced photosynthetic efficiency, and heightened metabolism of sugars and organic compounds [64,65,66]. These coordinated physiological changes enable temperate deciduous trees to optimize resource allocation, enhance protection against environmental stress, and adapt to variable climates. Consequently, the molecular and physiological strategies identified in this study hold broad applicability, offering valuable insights into how diverse temperate trees respond to and survive environmental variation.

## 4. Materials and Methods

### 4.1. Plant Material

**Figure 11:**
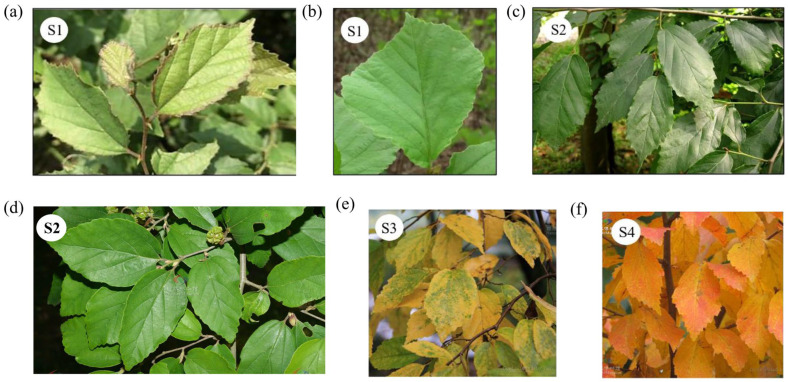
Leaf color variation in *Parrotia subaequalis* across four developmental stages. (**a**,**b**) Early spring (S1); (**c**,**d**) late spring to early summer (S2); (**e**) autumn transition with partial yellowing (S3); (**f**) fully yellow leaves in winter (S4).

To minimize the effects of phenotypic plasticity and environmental heterogeneity, we established a common garden experiment using *P. subaequalis* individuals collected from 14 different populations. Three-year-old seedlings were propagated through clonal grafting to ensure consistent genetic backgrounds under uniform growth conditions. Branches with distinct leaf coloration were monitored throughout the year. Based on long-term observations, we defined four representative discoloration stages: (S1) early spring (March–April), when leaves were bright green or displayed purple-red margins; (S2) late spring to early summer (May–June), when leaves appeared uniformly bottle-green; (S3) autumn (September–November), when approximately half the leaf surface turned yellow or red while the center remained green; and (S4) winter (December–January), when leaves were nearly completely yellow or red (Figure 11). Leaf color at each stage was quantified using a colorimeter.

### 4.2. Phenotypic Measurements

For each population, 20 healthy, pest/disease-free individuals were selected. From each individual, 10 mature leaves from the upper canopy were collected, ensuring intact petioles wherever feasible. Morphological traits were assessed and recorded immediately. Petiole length, lamina length, maximum lamina width, lamina width at 10% of length, lamina width at 90% of length, and number of principal veins were measured using a digital caliper (precision: 0.01 mm). Three replicate measurements were taken per trait, and the mean value was calculated [67].

### 4.3. Physiological Measurements

Chlorophyll content was determined following standard spectrophotometric procedures. Fresh leaf tissue (0.2 g) was cut it into pieces and placed in a mortar. A small amount of quartz sand and 80% acetone were added, and the tissue was ground until fully homogenized and decolorized. The homogenate was filtered and transferred to a 25 mL volumetric flask, which was brought to volume with 80% (*v*/*v*) acetone. Absorbance readings were taken at 663 nm and 645 nm using a UV-VIS spectrophotometer (Beijing Spectrometer General Co., Ltd, Beijing, China), with 80% acetone as the blank. Each sample was analyzed in triplicate. Chlorophyll a (Chl a), chlorophyll b (Chl b), total chlorophyll, and carotenoids were calculated based on absorbance values using Lambert–Beer’s law [68,69].

Chl a = (12.7 A_663_ − 2.59 A_645_) × V/(1000 × FW)

Chl b = (22.88 A_645_ − 4.76 A_663_) × V/(1000 × FW)

Total Chl = 0.01 × (20.21 × A_645_ + 8.02 × A_663_) × D/m

Carotenoid = 0.01 × [(1000A_470_ − 3.27C_a_ − 104C_b_)/229] × D/m

Here, A_663_ and A_645_ represent the absorbance values at their corresponding wavelengths, V denotes the volume of the extract in milliliters, and FW represents the fresh weight of the leaves in grams. D represents the dilution ratio, and m represents sample mass (g) in grams. The content of lutein was measured using high-performance liquid chromatography (HPLC) (Agilent series 1100, Agilent, Waldbronn, Germany) [70]. The content of D-glucose and sucrose was determined using a Shimadzu High-Performance Liquid Chromatograph SPD-20A/20AV UV-Vis detector. Chromatographic separation using an Agilent ZORBAX NH2 column (4.6 × 250 mm, 5 μm particle size). The column temperature was maintained at 40 °C, with a flow rate of 1 mL/min and an injection volume of 20 μL.

Photosynthetic parameters were measured using Li-6400 photosynthesis equipment (LI-COR, Lincoln, NE, USA). All parameters, including net photosynthetic rate and stomatal conductance, were determined under a light intensity of 700 μmol·m^−2^·s^−1^, and the CO_2_ concentration was 400 μmol/mol^−1^.

### 4.4. Transcriptome Sequencing and Analysis

Fresh leaf samples from each stage were collected, immediately frozen in liquid nitrogen, and stored at −80 °C in ultra-low temperature freezers. Three biological replicates were used for subsequent transcriptome profiling. According to the manufacturer’s instructions, total RNA for transcriptome sequencing was extracted using an RNAprep pure Plant Kit (Tiangen, Beijing, China). RNA quality was determined using a NanoDrop ND1000 spectrophotometer (NanoDrop Technologies, Wilmington, DE, USA) and further validated using the Agilent Bioanalyzer 2100 system (Agilent Technologies, Santa Clara, CA, USA).

After quantification and qualification of the RNA samples, cDNA libraries were constructed using the mRNA-Seq Sample Preparation Kit™ (Illumina, San Diego, CA, USA). The DNA yield and fragment size distribution of each library were determined using the Agilent Bioanalyzer. The cDNA libraries were sequenced on the Illumina High Sequencing platform using Sequencing by Synthesis technology. Linker sequences and low-quality reads were filtered out to obtain clean reads, which were then assembled using Trinity (v2.5.1) to generate a high-quality reference sequence. Transcript levels or gene expression were quantified using FPKM (Fragments Per Kilobase of transcript per Million fragments mapped). Gene function annotation was performed using several databases, including NR (NCBI), PFAM, Swiss-Prot, GO, KOG/COG, eggNOG4.5, and KEGG. Differentially expressed gene (DEG) identification was further performed using the DESeq2 R package [71] with criteria of |Log_2_(Fold Change)| > 2 and *q*-value ≤ 0.01. Gene Ontology (GO) analysis and KEGG pathway enrichment analysis were conducted using the ClusterProfile R package [72].

### 4.5. Metabolomic Analysis

Leaf samples from the S1 and S4 stages were collected for metabolite extraction and analysis, with each plant represented by three biological replicates. Fresh leaves were ground under liquid nitrogen, and 0.5 mL of 80% (*v*/*v*) methanol was added to each sample (0.1 g) and then vortexed. After incubation in an ice bath for 5 min, the samples were centrifuged at 15,000× *g* and 4 °C for 20 min. The supernatant was recovered, and water was added to dilute the methanol solution to 53% (*v*/*v*). Following a second centrifugation at 15,000× *g* and 4 °C for 20 min, the supernatant was collected for subsequent liquid chromatography tandem mass spectrometer (LC-MS/MS) analysis [73]. Metabolite identification and quantification followed the protocol by Chen et al. (2013) [74]. Partial least squares discriminant analysis (PLS–DA) was conducted using the identified metabolites. Metabolites showing significant differences in content were selected based on thresholds of variable importance in projection (VIP) ≥ 1 and fold change ≥ 2 or ≤0.5.

Raw LC-MS/MS data were processed to generate qualitative identifications and relative quantification of metabolites. Metabolite annotation was performed using the KEGG, HMDB and LIPIDMaps databases. The criteria for identifying differentially accumulated metabolites (DAMs) were set as VIP > 1, *p*-value < 0.05, and fold change > 1.5. Statistical analysis and pathway enrichment were conducted using the ClusterProfile R package [75].

### 4.6. Correlation Analysis of Transcriptome and Metabolome

The DEGs screened from the transcriptome analysis and the DAMs identified in the metabolome analysis were used for KEGG enrichment analysis to identify co-enriched biochemical pathways. The metabolites were classified based on their positive-ion response and negative-ion response.

### 4.7. qRT-PCR

To validate the transcriptome data, the relative expression levels of four genes identified through transcriptomic analysis were evaluated using qRT-PCR, with three biological replicates and three technical replicates. RNA extraction was performed using the Tiangen total RNA extraction kit (Tiangen, Beijing, China), and cDNA synthesis was carried out using the PrimeScript RT reagent Kit with gDNA Eraser (TaKaRa, Kyoto, Japan). Primers were designed based on target gene sequences retrieved from NCBI. qRT-PCR was conducted using AceQ qPCR SYBR Green Master mix (Vazyme). The relative expression levels were analyzed using the 2^−△△Ct^ method, with 18S-rRNA serving as the reference gene.

### 4.8. Data Analysis

Summary statistics (mean, standard deviation, maximum, minimum, and range) were calculated for each trait in Excel 2010. Within- and between-population variation was quantified via the coefficient of variation (CV = standard deviation/mean × 100%). Variance components within and between populations were calculated following Ge et al. (1988) [76]. To assess population-specific effects on leaf traits, a Poisson regression model with log-link function was utilized due to the non-normal distribution of the data.

Data analysis and graphical representation were performed using R software (version R 3.5.0) [77]. The correlations between differentially expressed genes (DEGs) and differentially accumulated metabolites (DAMs) were assessed using Pearson’s correlation coefficients. KEGG enrichment analysis was visualized using the R package “ggplot2” [78]. Statistical significance was evaluated using a *t*-test, with significance levels denoted by asterisks as follows: *** *p* < 0.001, ** *p* < 0.01, * *p* < 0.05, while non-significant results were marked as ‘ns’. Error bars represent the standard error of the mean (SEM).

## 5. Conclusions

This study identified key physiological and molecular pathways underlying leaf color change in *Parrotia subaequalis*, while also documenting substantial morphological variation across its natural range. Phenotypic comparisons among 14 populations revealed significant differences in leaf traits—particularly in petiole length, lamina dimensions, and leaf area—suggesting high plasticity and potential for local adaptation. Focusing on the YX population, which exhibited consistent yellowing during late developmental stages, integrated transcriptomic and metabolomic analyses revealed that starch and sucrose metabolism, carbon metabolism, and photosynthesis were central to the observed pigment transitions. Yellow leaves displayed reduced pigment content and photosynthetic activity, alongside increased D-glucose accumulation. These findings enhance understanding of how both morphological diversity and molecular regulation contribute to visible phenotypic traits in rare plants. The results also provide a foundation for future research linking population-level variation with underlying molecular mechanisms in *P. subaequalis* and related species. This work highlights the value of integrating ecological, physiological, and molecular data in the conservation and sustainable use of endangered plant taxa.

## Figures and Tables

**Table 1:** Details of sample locations and sizes of 14 populations of *Parrotia subaequalis*.

Province	Sample Location	Population Code	Latitude (°N)	Longitude (°E)	Altitude (m)	Sample Size	Mountain	Autumn Color
Zhejiang	Ningbo	NB	29.68	121.03	988	23	Tianmu	Red
	Changhua (Hangzhou)	CH	30.16	119.18	864	42		Red
	Anji (Huzhou)	AJ	30.38	119.4	820	30		Purple
Jiangsu	Yixing	YX	31.23	119.73	252	39		Yellow
Anhui	Jixi	JX	30.2	118.88	684	23	Dabie	Purple
	Jingde	JD	30.41	118.58	653	31		Red
	Yuexi (Huangwei)	YXI	31.1	116.31	449	25		Pink
	Yuexi (Hetu)	YXII	31.81	116.03	313	29		Pink
	Yuexi (Zhubo)	YXIII	31.03	116.48	583	31		Pink
	Shucheng (Lu’an)	SC	31.06	116.55	584	29		Red
	Tongcheng (Anqing)	TC	31.08	116.85	270	24		Red
	Jinzhai (Lu’an)	JZ	31.2	115.9	450	24		Purple
	Huoshan (Lu’an)	HS	31.25	116.01	530	27		Pink
Henan	Xinyang	XY	31.45	115.26	192	28		Red

**Table 2:** Geographical locations and their climate conditions for 14 populations of *P. subaequalis*.

Sample Location	Latitude (N)	Longitude (E)	Altitude (m)	Mean Soil Depth (cm)	Average Annual Precipitation (mm)	Average Annual Air Temperature (°C)
CH	30°10′	119°11′	864	25.5	1123.6	12.2
AJ	30°23′	119°24′	820	11	1220.1	15
NB	29°41′	121°02′	988	20	1110.4	11.1
YX	31°14′	119°44′	252	27	1294.6	14.1
JX	30°12′	118°53′	684	39	1357.2	12.1
JD	30°25′	118°35′	653	21	1286.1	14.1
TC	31°05′	116°51′	270	34	1290.5	14
HS	31°15′	116°01′	530	12	1351.3	14.6
SC	31°4′	116°33′	584	28	1171.8	12.8
JZ	31°12′	115°54′	450	42	1419.9	15.9
YXIII	31°02′	116°29′	583	26	1296.4	14.6
YXII	30°49′	116°02′	313	24	1290.4	14.6
YXI	31°06′	116°19′	449	25	1290.4	14.6
XY	31°27′	115°16′	192	26	1380.5	15.8

**Table 3:** Analysis of Variance (ANOVA) of phenotypic traits of leaf among and within *P. subaequalis* populations.

Traits	Mean Square			*F*-Value	
	Among Populations	Within Populations	Random Error	Among Populations	Within Populations
Petiole length	0.397	0.117	0.017	23.152 **	7.718 **
Lamina length	37.264	5.133	0.888	41.955 **	8.529 **
Maximum lamina width	4.063	1.369	0.842	4.828 **	2.631 **
Lamina width at 10% of length	3.087	0.412	0.208	14.804 **	2.703 **
Lamina width at 90% of length	2.827	0.526	0.748	3.779 **	2.469 **
Number of principal veins	66.422	15.673	2.138	31.060 **	9.153 **

** *p* < 0.01.

**Table 4:** Variance analysis of phenotypic traits among 14 *P. subaequalis* populations.

Traits	Populations														
	CH	AJ	NB	YX	JX	JD	TC	HS	SC	JZ	YXIII	YXⅠI	YXI	XY	Mean
Petiole length/%	22.11	27.24	15.98	22.76	25.49	28.16	26.67	25.63	35.91	22.57	29.02	33.93	56.06	22.78	28.58
Lamina length/%	17.61	17.75	9.46	20.91	17.10	19.61	22.56	14.07	24.45	20.40	22.61	22.45	19.66	20.06	19.13
Maximum lamina width/%	18.35	17.43	12.74	20.12	17.86	52.80	25.24	13.92	25.86	21.08	21.95	24.75	19.98	21.17	22.47
Lamina width at 10% of length/%	46.64	33.17	35.63	40.40	45.49	47.84	52.72	33.93	71.97	56.95	42.28	51.27	55.62	71.63	47.22
Lamina width at 90% of length/%	26.97	24.10	18.14	32.60	22.92	81.70	32.03	15.11	54.76	34.07	39.46	38.19	91.46	27.60	39.35
Number of principal veins/%	10.98	15.81	8.94	13.18	12.10	13.77	12.19	14.92	14.46	11.12	17.90	12.70	11.81	11.66	13.07
Leaf area	33.98	29.47	46.57	44.69	38.58	37.92	50.23	40.56	53.55	39.40	45.76	47.46	35.13	41.06	41.74
Leaf weight	39.50	36.69	59.15	57.14	45.20	48.93	54.32	50.74	54.71	54.54	72.13	55.55	45.45	50.72	51.74
Mean/%	27.01	25.21	25.83	31.47	28.09	41.34	34.49	26.11	41.96	32.51	36.38	35.78	41.89	33.33	32.91

**Table 5:** The mean value, standard deviation, and multiple comparison of phenotypic traits of 14 populations of *P. subaequalis.* Values are Mean ± SD (*n* = 100).

Population	Mean ± SD							
	LP	LL	MWL	WL (0.1)	WL (0.9)	NPV	LA	LW
CH	0.524 ± 0.116	5.470 ± 0.963	3.239 ± 0.594	0.943 ± 0.440	1.433 ± 0.387	11.780 ± 1.293	11.984 ± 4.073	0.081 ± 0.032
AJ	0.578 ± 0.157	5.716 ± 1.014	3.264 ± 0.569	1.266 ± 0.420	1.573 ± 0.379	11.010 ± 1.714	13.901 ± 4.097	0.109 ± 0.040
NB	0.553 ± 0.088	4.637 ± 0.439	3.090 ± 0.394	1.230 ± 0.438	1.837 ± 0.333	11.367 ± 1.016	10.546 ± 4.912	0.071 ± 0.042
YX	0.627 ± 0.143	5.916 ± 1.237	3.293 ± 0.663	1.299 ± 0.525	1.667 ± 0.543	10.905 ± 1.437	14.059 ± 6.284	0.077 ± 0.044
JX	0.510 ± 0.130	5.586 ± 0.955	3.364 ± 0.601	1.132 ± 0.515	1.709 ± 0.392	11.833 ± 1.432	11.425 ± 4.408	0.073 ± 0.033
JD	0.455 ± 0.128	4.528 ± 0.888	2.941 ± 1.552	0.961 ± 0.460	1.364 ± 1.114	10.140 ± 1.397	9.425 ± 3.574	0.047 ± 0.023
TC	0.538 ± 0.143	6.148 ± 1.387	3.387 ± 0.885	1.088 ± 0.574	1.349 ± 0.432	13.428 ± 1.636	15.430 ± 7.751	0.081 ± 0.044
HS	0.387 ± 0.099	5.467 ± 0.769	3.133 ± 0.436	1.030 ± 0.349	1.997 ± 0.302	10.933 ± 1.623	11.121 ± 4.511	0.067 ± 0.034
SC	0.443 ± 0.159	5.156 ± 1.261	3.127 ± 0.808	0.588 ± 0.423	0.765 ± 0.419	10.830 ± 1.566	11.237 ± 6.018	0.053 ± 0.029
JZ	0.491 ± 0.111	5.356 ± 1.093	3.294 ± 0.694	1.073 ± 0.611	1.489 ± 0.507	12.586 ± 1.399	11.434 ± 4.506	0.066 ± 0.036
YXIII	0.403 ± 0.117	4.356 ± 0.985	2.513 ± 0.552	0.948 ± 0.401	1.127 ± 0.445	10.186 ± 1.823	10.782 ± 4.934	0.061 ± 0.044
YXII	0.472 ± 0.160	5.027 ± 1.129	2.909 ± 0.720	1.096 ± 0.562	1.461 ± 0.558	10.731 ± 1.363	10.327 ± 4.902	0.081 ± 0.045
YXI	0.502 ± 0.281	5.197 ± 1.022	3.039 ± 0.607	0.948 ± 0.527	1.354 ± 1.238	11.480 ± 1.356	10.128 ± 3.558	0.066 ± 0.030
XY	0.564 ± 0.129	5.235 ± 1.050	3.146 ± 0.666	1.087 ± 0.778	1.568 ± 0.433	12.509 ± 1.458	11.139 ± 4.574	0.069 ± 0.035
*p*-value	<0.001	<0.001	<0.001	<0.001	<0.001	<0.001	<0.001	<0.001
Mean	0.5	5.27	3.12	1.05	1.48	11.41	11.63	0.07

## Data Availability

Data will be provided upon request.

## Supplementary Materials

The following supporting information can be downloaded at: https://www.mdpi.com/article/10.3390/plants14152345/s1, Figure S1: The transcription factors regulate the pigment change of leaves. Figure S2: The expression level of *PSA, PSB28, HXK1* and *SCO2*. Figure S3: The summary figure linking transcript, metabolite and pigment shifts. Tables S1–S6: Generalized linear regression analysis examining the effects of populations with its petiole length, lamina length, maximum lamina width, lamina width at 10% of length, lamina width at 90% of length, and number of principal veins, Table S7: The quality of clean reads, Table S8–S14: The annotation of genes in four developmental stage of leaves.
