# Supplementary material for: The Scarlet Alchemy of Survival: Integrated Transcriptomic and Metabolomic Analysis of Leaf Coloration in Endangered Parrotia subaequalis
Source: Plants (Basel). 2025 Jul 29;14(15):2345. doi: 10.3390/plants14152345 (PMC12348515; doi:10.3390/plants14152345)
Supplement: Supplementary file 1 [file plants-14-02345-s001.zip › Figure S2.pdf]

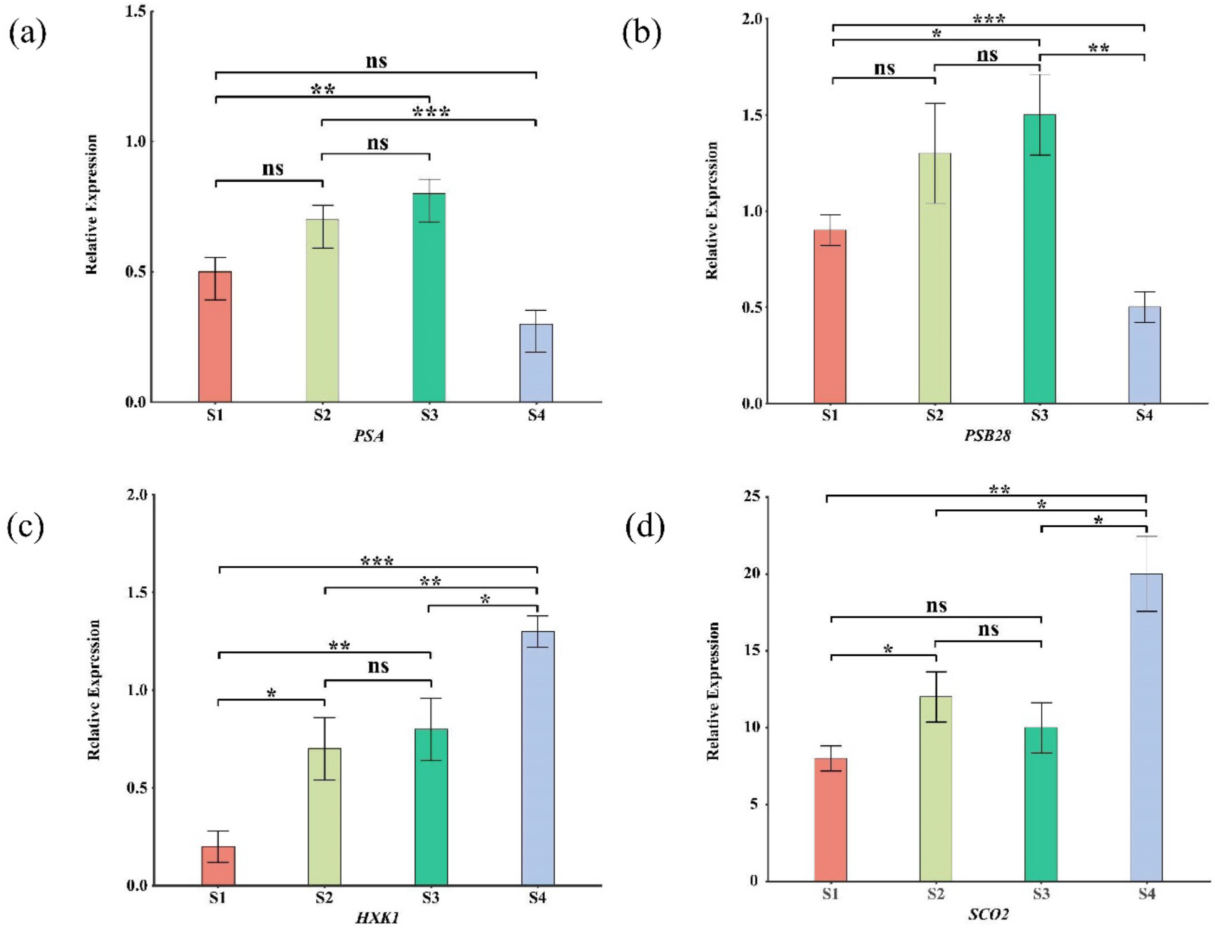

Figure S2. The expression level of *PSA*, *PSB28*, *HXK1* and *SCO2*. Data represent the means ± SEM from at least three biological replicates; ns, not significant, **P* <0.05, ***P* <0.01, ****P<0.001*, by *t*-test.
