# Supplementary material for: The Scarlet Alchemy of Survival: Integrated Transcriptomic and Metabolomic Analysis of Leaf Coloration in Endangered Parrotia subaequalis
Source: Plants (Basel). 2025 Jul 29;14(15):2345. doi: 10.3390/plants14152345 (PMC12348515; doi:10.3390/plants14152345)
Supplement: Supplementary file 1 [file plants-14-02345-s001.zip › Supplementary_Table_S1.pdf]

Table S1. Generalized Linear regression analysis examining the effects of populations with its petiole length. *P*-values < 0.05 are boldfaced. The sample size n=200

Population	Estimate	SE	*z*	*P*
Intercept	27.240	5.219	5.219	**1.80e-07*****
CH	-5.130	7.025	-0.73	0.46524
HS	-1.610	7.271	-0.221	0.82476
JD	0.920	7.443	0.124	0.90163
JX	-1.750	7.261	-0.241	0.80956
JZ	-4.670	7.058	-0.662	0.50817
NB	-11.260	6.574	-1.713	0.08676
SC	8.670	7.947	1.091	0.27526
TC	-0.570	7.342	-0.078	0.93812
XY	-4.460	7.072	-0.631	0.52829
YX	-4.480	7.071	-0.634	0.52636
YXI	28.820	9.127	3.158	0.00159
YXII	6.690	7.821	0.855	0.39234
YXIII	1.780	7.501	0.237	0.81241
