# Supplementary material for: The Scarlet Alchemy of Survival: Integrated Transcriptomic and Metabolomic Analysis of Leaf Coloration in Endangered Parrotia subaequalis
Source: Plants (Basel). 2025 Jul 29;14(15):2345. doi: 10.3390/plants14152345 (PMC12348515; doi:10.3390/plants14152345)
Supplement: Supplementary file 1 [file plants-14-02345-s001.zip › Supplementary_Table_S2.pdf]

Table S2. Generalized Linear regression analysis examining the effects of populations with its lamina length. *P*-values < 0.05 are boldfaced. The sample size n=200

**Population**	**Estimate**	**SE**	***z***	***P***
Intercept	17.75	4.213	4.213	**2.52e-05** ***
CH	-0.14	5.946	-0.024	0.981
HS	-3.68	5.641	-0.652	0.514
JD	1.86	6.112	0.304	0.761
JX	-0.65	5.903	-0.11	0.912
JZ	2.65	6.177	0.429	0.668
NB	-8.29	5.216	-1.589	0.112
SC	6.7	6.496	1.031	0.302
TC	4.81	6.349	0.758	0.449
XY	2.31	6.149	0.376	0.707
YX	3.16	6.218	0.508	0.611
YXI	1.91	6.116	0.312	0.755
YXII	4.7	6.34	0.741	0.459
YXIII	4.86	6.353	0.765	0.444
