# Supplementary material for: The Scarlet Alchemy of Survival: Integrated Transcriptomic and Metabolomic Analysis of Leaf Coloration in Endangered Parrotia subaequalis
Source: Plants (Basel). 2025 Jul 29;14(15):2345. doi: 10.3390/plants14152345 (PMC12348515; doi:10.3390/plants14152345)
Supplement: Supplementary file 1 [file plants-14-02345-s001.zip › Supplementary_Table_S3.pdf]

Table S3. Generalized Linear regression analysis examining the effects of populations with its maximum lamina width. *P*-values < 0.05 are boldfaced. The sample size n=200

**Population**	**Estimate**	**SE**	***z***	***P***
Intercept	17.43	4.175	4.175	**2.98e-05** ***
CH	0.92	5.982	0.154	0.878
HS	-3.51	5.599	-0.627	0.531
JD	35.37	8.38	4.221	**2.44e-05** ***
JX	0.43	5.941	0.072	0.942
JZ	3.65	6.206	0.588	0.556
NB	-4.69	5.493	-0.854	0.393
SC	8.43	6.58	1.281	0.2
TC	7.81	6.532	1.196	0.232
XY	3.74	6.213	0.602	0.547
YX	2.69	6.128	0.439	0.661
YXI	2.55	6.116	0.417	0.677
YXII	7.32	6.495	1.127	0.26
YXIII	4.52	6.275	0.72	0.471
