# Supplementary material for: The Scarlet Alchemy of Survival: Integrated Transcriptomic and Metabolomic Analysis of Leaf Coloration in Endangered Parrotia subaequalis
Source: Plants (Basel). 2025 Jul 29;14(15):2345. doi: 10.3390/plants14152345 (PMC12348515; doi:10.3390/plants14152345)
Supplement: Supplementary file 1 [file plants-14-02345-s001.zip › Supplementary_Table_S4.pdf]

Table S4. Generalized linear regression analysis examining the effects of populations with its lamina width at 10% of length. *P*-values < 0.05 are boldfaced. The sample size n=200

**Population**	**Estimate**	**SE**	*z*	***P***
Intercept	33.17	5.759	5.759	**8.44e-09** ***
CH	13.47	8.934	1.508	0.13161
HS	0.76	8.191	0.093	0.926079
JD	14.67	9.001	1.63	0.103123
JX	12.32	8.869	1.389	0.164802
JZ	23.78	9.493	2.505	**0.012246** *
NB	2.46	8.295	0.297	0.766788
SC	38.8	10.254	3.784	**0.000154** ***
TC	19.55	9.268	2.109	**0.294275** *
XY	38.46	10.237	3.757	**0.000172** ***
YX	7.23	8.577	0.843	0.399271
YXI	22.45	9.423	2.383	**0.017195** *
YXII	18.1	9.189	1.97	**0.048870** *
YXIII	9.11	8.686	1.049	0.294275
