# Supplementary material for: The Scarlet Alchemy of Survival: Integrated Transcriptomic and Metabolomic Analysis of Leaf Coloration in Endangered Parrotia subaequalis
Source: Plants (Basel). 2025 Jul 29;14(15):2345. doi: 10.3390/plants14152345 (PMC12348515; doi:10.3390/plants14152345)
Supplement: Supplementary file 1 [file plants-14-02345-s001.zip › Supplementary_Table_S5.pdf]

Table S5. Generalized Linear regression analysis examining the effects of populations with its lamina width at 90% of length. *P*-values < 0.05 are boldfaced. The sample size n=200

Population	Estimate	SE	*z*	*P*
Intercept	24.1	4.909	4.909	**9.15e-07** ***
CH	2.87	7.146	0.402	0.687975
HS	-8.99	6.262	-1.436	0.15109
JD	57.6	10.286	5.6	**2.14e-08** ***
JX	-1.18	6.857	-0.172	0.863371
JZ	9.97	7.627	1.307	0.191141
NB	-5.96	6.499	-0.917	0.359126
SC	30.66	8.88	3.453	**0.000555** ***
TC	7.93	7.492	1.058	0.289844
XY	3.5	7.19	0.487	0.626422
YX	8.5	7.53	1.129	0.258971
YXI	67.36	10.75	6.266	**3.70e-10** ***
YXII	14.09	7.892	1.785	0.074219.
YXIII	15.36	7.972	1.927	0.054025.
