# Supplementary material for: The Scarlet Alchemy of Survival: Integrated Transcriptomic and Metabolomic Analysis of Leaf Coloration in Endangered Parrotia subaequalis
Source: Plants (Basel). 2025 Jul 29;14(15):2345. doi: 10.3390/plants14152345 (PMC12348515; doi:10.3390/plants14152345)
Supplement: Supplementary file 1 [file plants-14-02345-s001.zip › Supplementary_Table_S6.pdf]

Table S6. Generalized Linear regression analysis examining the effects of populations with its number of principal veins. *P*-values < 0.05 are boldfaced. The sample size n=200

**Population**	**Estimate**	**SE**	***z***	***P***
Intercept	15.81	3.976	3.976	**7e-05** ***
CH	-4.83	5.176	-0.933	0.351
HS	-0.89	5.543	-0.161	0.872
JD	-2.04	5.439	-0.375	0.708
JX	-3.71	5.283	-0.702	0.483
JZ	-4.69	5.189	-0.904	0.366
NB	-6.87	4.975	-1.381	0.167
SC	-1.35	5.502	-0.245	0.806
TC	-3.62	5.292	-0.684	0.494
XY	-4.15	5.241	-0.792	0.428
YX	-2.63	5.384	-0.488	0.625
YXI	-4	5.255	-0.761	0.447
YXII	-3.11	5.34	-0.582	0.56
YXIII	2.09	5.806	0.36	0.719
