# Supplementary figures and images for: The Scarlet Alchemy of Survival: Integrated Transcriptomic and Metabolomic Analysis of Leaf Coloration in Endangered Parrotia subaequalis
Source: Plants (Basel). 2025 Jul 29;14(15):2345. doi: 10.3390/plants14152345 (PMC12348515; doi:10.3390/plants14152345)

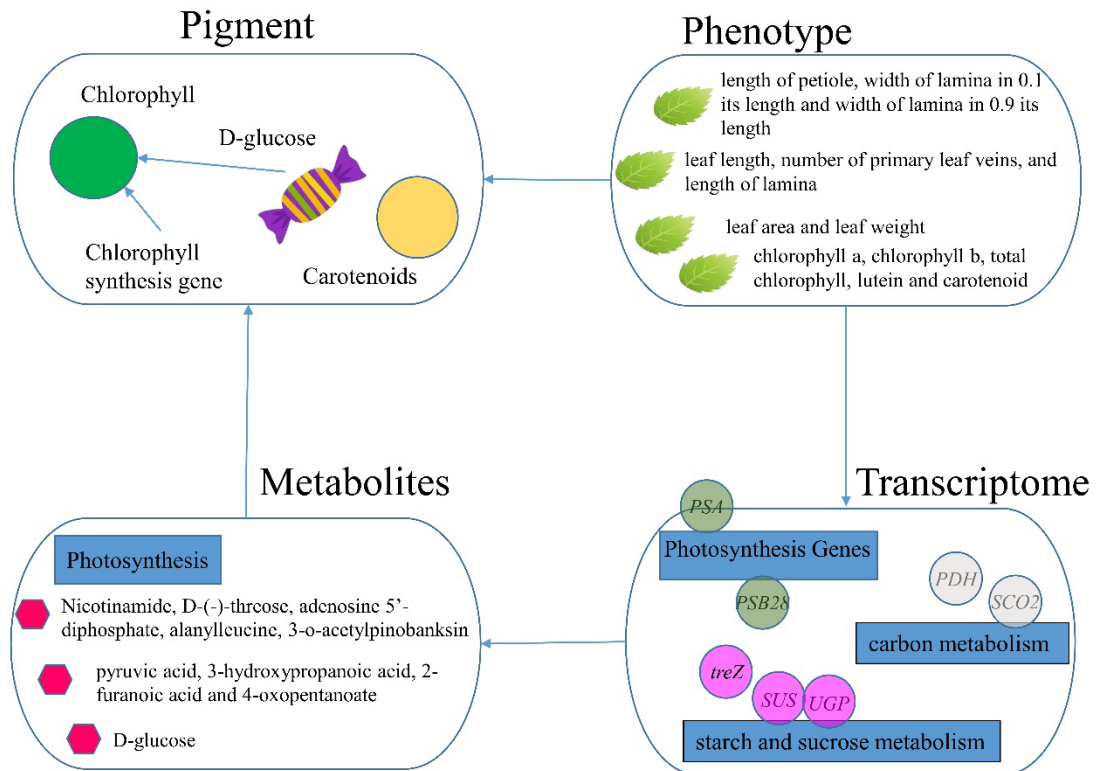

**Figure S3.** The summary figure linking transcript, metabolite and pigment shifts.

Supplement: Supplementary file 1 [file plants-14-02345-s001.zip › Figure S3.pdf]

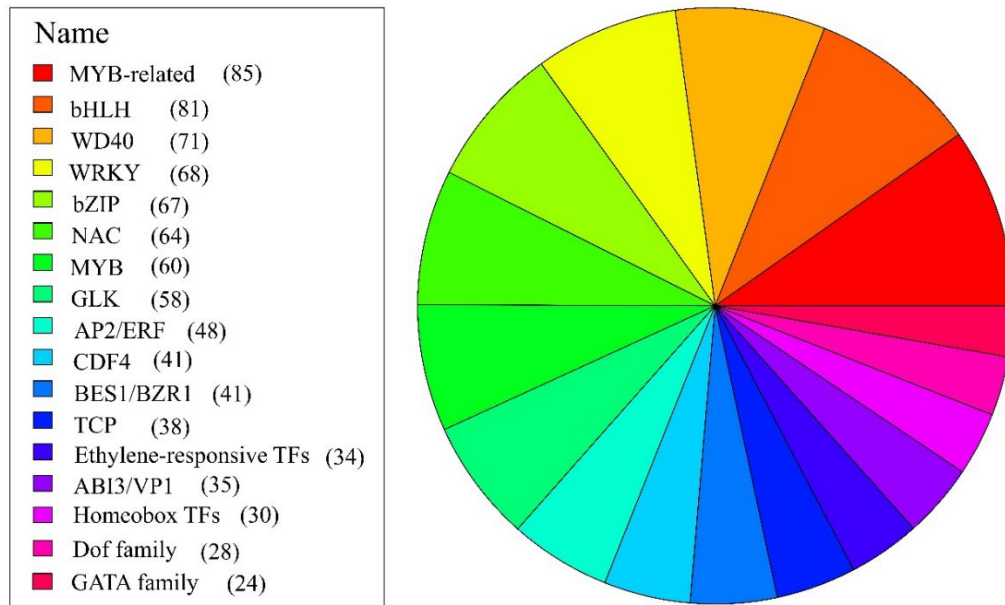

**Figure S1.** The transcription factors regulate the pigment change of leaves.

Supplement: Supplementary file 1 [file plants-14-02345-s001.zip › Figure S1.pdf]
